# How the Spectre of Societal Homogeneity Undermines Equitable Healthcare for Refugees: Comment on "Defining and Acting on Global Health: The Case of Japan and the Refugee Crisis"

**DOI:** 10.15171/ijhpm.2016.139

**Published:** 2016-10-17

**Authors:** Oliver Razum, Judith Wenner, Kayvan Bozorgmehr

**Affiliations:** ^1^Department of Epidemiology and International Public Health, School of Public Health, Bielefeld University, Bielefeld, Germany.; ^2^Department of General Practice and Health Services Research, University Hospital Heidelberg, Heidelberg, Germany.

**Keywords:** Germany, Refugee, Access to Healthcare, Homogeneity, Equity

## Abstract

Recourse to a purported ideal of societal homogeneity has become common in the context of the refugee reception crisis – not only in Japan, as Leppold et al report, but also throughout Europe. Calls for societal homogeneity in Europe originate from populist movements as well as from some governments. Often, they go along with reduced social support for refugees and asylum seekers, for example in healthcare provision. The fundamental right to health is then reduced to a citizens’ right, granted fully only to nationals. Germany, in spite of welcoming many refugees in 2015, is a case in point: entitlement and access to healthcare for asylum seekers are restricted during the first 15 months of their stay. We show that arguments brought forward to defend such restrictions do not hold, particularly not those which relate to maintaining societal homogeneity. European societies are not homogeneous, irrespective of migration. But as migration will continue, societies need to invest in what we call "globalization within." Removing entitlement restrictions and access barriers to healthcare for refugees and asylum seekers is one important element thereof.

The idea that societies should be, remain, or become more homogenous is a recurring phenomenon. This shows for example in the policy of a country towards refugees and asylum seekers. Japan, as Claire Leppold and colleagues illustrate, is a case in point.^1^ Japan receives a small number of asylum applications, compared to many European countries; and it has accepted only a minute proportion in the past years (fewer than one in 200). Leppold et al suggest that cultural and historical factors determine the ways in which the country perceives and acts, citing the purportedly Japanese ideal of societal homogeneity.

Yet calls for societal homogeneity have become a characteristic not only of Japan. Similar calls can now be heard throughout Europe in the context of the refugee reception crisis – either from governments such as those of Poland and Hungary,^2^ or from populist, xenophobic or right-wing political movements, for example in Germany.^3^ And these calls go beyond the question of refuges and asylum seekers. They reach farther, right into economic and foreign politics. As “*The Economist*” put it on the cover of its July 30, 2016, edition: “The new political divide” is no longer left v right but “open v closed.” Hence, Japan’s refugee policy fits seamlessly into a global trend favouring closed societies and restrictive migration policies.

## Access to Healthcare for Refugees: “Exclusion Within”

When it comes to refugees and asylum seekers, “open v closed” often translates to “them v us,” affecting social policies such as entitlements to healthcare.^4^ As Gorik Ooms points out, the right to the best possible health is then reduced to a citizens’ right, granted to people in a country according to their residency status; it no longer is a universal human right, granted to everybody in need in the respective country.^5^ This degradation of a fundamental right can be observed in a number of European countries, as the comparative work of the MIPEX health strand researchers shows.^6^

The idea of homogeneity might be underlying the non-acceptance of refugees and asylum seekers in Japan. High numbers of refugees and asylum seekers, however, are certainly not indicating the absence of the ideal of homogeneity. It rather seems that while in the case of Japan it is leading to “geographical exclusion,” in Europe it is translating into “societal exclusion” or “exclusion within.” Germany, lauded for its welcoming attitude towards refugees in 2015,^2^ is a good example. High numbers of asylum seekers entering Germany are masking the fact that Germany is ranked only in the lower half of the MIPEX list (place 23 out of 38) due to comparatively exclusionist welfare policies. Regular immigrants to Germany, such as work migrants, have the same entitlements to healthcare coverage through the statutory health insurance as the majority population. This, however, is not the case for refugees and asylum seekers. According to paragraph 4 of the Asylum Seekers’ Benefit Act (AsylbLG), their entitlement is restricted to the care for acute pain, pregnancy and child birth, as well as immunizations in the first 15 months. Additional care can be financed only on a case-by-case basis according to paragraph 6 of AsylbLG. In practice, there are additional barriers for refugees needing to access care. Care provision is regulated in different ways by the communities; it is further complicated by different regulations at federal state levels and by differences in knowledge of the physicians on how entitlement restrictions can be circumvented on a case-by-case basis.^7,8^ Thus, in Germany, entitlement and access to healthcare is severely restricted for asylum seekers and refugees, excluding them not physically but socially.

Underlying these restrictions are two ideas: one, entitlement of healthcare should not become a “pull factor” for asylum seekers; and two, the cost of their healthcare provision should be contained. So far, there is no scientific evidence supporting the first idea.^9^ The value of the idea of pull factors for analysing individual decisions to migrate is limited. The actions of refugees and asylum seekers are determined by a complex web of contextual factors and individual characteristics, with most drivers of migration being found in the countries of origin.^10-13^ It seems cynical to assume that a person would leave a bombarded Syrian city with the primary aim of seeking healthcare in Germany; it rather is an act of seeking protection from mortal peril.

The second idea has been soundly refuted: restricted entitlements to healthcare are associated not with savings, but actually with higher healthcare expenditure.^7^ The underlying mechanisms have not yet been thoroughly studied, not the least because the necessary data are not being collected.^14^ Probably, the restrictions lead to delay in treatment, with later interventions that are more complex compared to early treatment or preventive measures. In extreme cases, the restrictions may lead to delayed treatment of life-threatening conditions.^15^ Even if catastrophes do not occur, barriers in access to healthcare are easily interpreted as a sign of non-welcome by the host society. Health, and by implication access to healthcare, however, constitute important resources for social participation and integration of immigrants and asylum seekers.^16^ Restricting it is likely to negatively affect their social integration.

## The Illusion of Homogeneity

This mind-set of “open v closed” follows the idea of societal homogeneity. Why is the idea of homogeneity of a society so compelling and tempting? A strong underlying motive is fear. As the German sociologist Heinz Bude points out, fear – irrespective of its origin – is often projected on what is unknown or perceived as different.^17^ The target of such projections often are minority groups such as immigrants or refugees. Thus, fear in the majority population, eg, of economic exclusion or loss of status, is projected on groups which are as well in desperate need of inclusive measures (of which equitable access to healthcare is just one example).

In fact, the concept of a homogeneous society is a mirage, a spectre. No society will ever be homogeneous, and there never existed a homogeneous society. Human history is a history of constant and large-scale migration, and thus, a history of unceasing genetic exchange.^18^ From a long-term perspective, societal heterogeneity has been normal, and in all likelihood an important determinant of human development. We tend to focus more on a short-term-perspective, and thereby on risks associated with migration.

Many societies, including those of the western industrialised countries, pretended to be homogeneous. But they never were,^19^ and the attempts at pretending created nightmarish situations for those who did not fit in. Didier Eribon, in his sociologically informed autobiographical work “Return to Reims,” describes memorably the differences in social class, education, religion, political as well as sexual orientation hidden behind the façade of a seemingly homogeneous white society in France in the second half of the 20th century – and the suffering this caused for members of this society who were constructed as not belonging.^20^ Since then, society at large, and healthcare services in particular, have been learning to adapt to the needs of different population groups. However, this remains an ongoing challenge, irrespective of immigration, as the situation eg, of transgender people shows.^21^

## Conclusions

We draw two main conclusions from discussing the paper by Leppold et al. First, societies will need to invest in what could be called a “globalization within”: Society members need to appreciate that societies are not homogeneous – they never truly were, and they never will be. Migration is a global phenomenon that will continue to impact on individual life courses as well as on societies as a whole. Immigration and offering refuge to asylum seekers is likely to add diversity to societies. In this respect, a new self-assertion is required: But this has been happening throughout human history. We need to (re-)appreciate that heterogeneity is a challenge – with migration being one of several drivers of heterogeneity and change –, but that accommodating it is a prerequisite for progress and development.

Second, societies will need to organize in such a way that all members can achieve, and maintain, a feeling of belonging.^13^ After the economic crisis of the 1930s, the welfare state was perceived as the road towards a self-assertive society free of fear.^17^ Today, a global perspective needs to be added even to seemingly domestic questions.^22^ The feeling of belonging must apply equally to long-established members of societies as well as to new ones such as asylum seekers.^13,20^ A precondition for belonging in diverse societies is equity with regard to fundamental rights. Enjoying equitable access to healthcare is one small aspect of this, but an important one. Hence, entitlement restrictions for refugees and asylum seekers should be abolished and other access barriers removed.

## Ethical issues

Not applicable.

## Competing interests

Authors declare that they have no competing interests.

## Authors’ contributions

All authors contributed to the text, read, and approved the final manuscript.

## Authors’ affiliations

^1^Department of Epidemiology and International Public Health, School of Public Health, Bielefeld University, Bielefeld, Germany. ^2^Department of General Practice and Health Services Research, University Hospital Heidelberg, Heidelberg, Germany.
